# Disinfection of human skin allografts in tissue banking: a systematic review report

**DOI:** 10.1007/s10561-016-9569-2

**Published:** 2016-08-13

**Authors:** C. Johnston, J. Callum, J. Mohr, A. Duong, A. Garibaldi, N. Simunovic, O. R. Ayeni, on behalf of the Bioburden Steering Committee and Skin Working group, Amber Appleby, Scott Brubaker, Jeannie Callum, Graeme Dowling, Ted Eastlund, Margaret Fearon, Marc Germain, Cynthia Johnston, Ken Lotherington, Ken McTaggart, Jim Mohr, Jutta Preiksaitis, Michael Strong, Martell Winters, Kimberly Young, Jie Zhao, Jeannie Callum, Robert Cartotto, Ian Davis, Ted Eastlund, Paul Gratzer, Cynthia Johnston, Lisa Merkley, Jim Mohr

**Affiliations:** 1 Capital Health Regional Tissue Bank, 5788 University Avenue, Room 431 Mackenzie Building, Halifax, NS B3H 1V7 Canada; 2 Sunnybrook Health Sciences Centre Blood and Tissue Bank, 2075 Bayview Ave., Room B2 04, Toronto, ON M4N 3M5 Canada; 3 Canadian Blood Services, 270 John Savage Ave., Dartmouth, NS B3B 0H7 Canada; 4 Department of Surgery, McMaster University, 293 Wellington St. N, Suite 110, Hamilton, ON L8L 8E7 Canada; 5 McMaster University Medical Centre, 1200 Main St W, Room 4E15, Hamilton, ON L8N 3Z5 Canada

**Keywords:** Skin allografts, Tissue donation, Tissue banking, Bioburden, Skin decontamination

## Abstract

The use of skin allografts to temporarily replace lost or damaged skin is practiced worldwide. Naturally occurring contamination can be present on skin or can be introduced at recovery or during processing. This contamination can pose a threat to allograft recipients. Bacterial culture and disinfection of allografts are mandated, but the specific practices and methodologies are not dictated by standards. A systematic review of literature from three databases found 12 research articles that evaluated bioburden reduction processes of skin grafts. The use of broad spectrum antibiotics and antifungal agents was the most frequently identified disinfection method reported demonstrating reductions in contamination rates. It was determined that the greatest reduction in the skin allograft contamination rates utilized 0.1 % peracetic acid or 25 kGy of gamma irradiation at lower temperatures.

**Electronic supplementary material:**

The online version of this article (doi:10.1007/s10561-016-9569-2) contains supplementary material, which is available to authorized users.

## Introduction

Skin banking is a process in which skin grafts are recovered from a cadaveric donor and stored (banked) for future use. Health Canada’s definition of “banked”, with respect to cells and tissues, are processed cells and tissues that have been determined safe for transplantation, and are stored by the source establishment in its inventory and are available for distribution or transplantation (Government of Canada 2013). The *contamination rate* represents the proportion of tissues with bacterial or fungal contamination at different points during graft processing, and the *bioburden* denotes the quantity of organisms in each sample. Bioburden reduction (disinfection) is defined as a process or intervention applied to skin grafts following recovery, which reduces bacteria and/or fungal bioburden. Reduction due to antimicrobial intervention can be assessed qualitatively in relation to changes in contamination rate or quantitatively by accurately determining the bioburden load before and after an intervention. Assessment of the effects of bioburden reduction processes on tissue viability and structural integrity are also critical for patient outcomes.

With the establishment of a number of tissue banks worldwide, there have been an increasing number of different practices reported for tissue disinfection. Increasing the storage period of skin is known to increase the probability of contamination (Gala et al. 1997). To reduce this, tissue banks strive to (1) minimize bioburden, (2) eliminate virulent organisms, and (3) maintain cell viability to contribute to optimal patient outcomes.

## Methods

### Information sources and search

The search strategy was developed by the Skin Processing and Validation Subgroup (through JM) and an information specialist. The search was applied to electronic databases MEDLINE and EMBASE from 1988 to July 7, 2014 using the following headings and text words: “skin,” “derm*,” “dermatoplast,” “allograft,” “anti-bacterial,” “anti-fungal,” “sterilization,” and “tissue banking”. The search included publications in English and excluded animal studies, case reports and conference abstracts. Two additional reviewers (AG and AD) performed a second search using the original search strategy to include publications from July 7, 2014 up to March 6, 2015. The full search strategy is shown in Online Resource 1.

### Study selection

Four reviewers (JM, SM, CP, PG) independently screened each of the citations in duplicate to identify studies that met all of the following inclusion criteria: (1) evaluated human skin (2) evaluated a method to reduce contamination rates, and (3) evaluated bioburden as an outcome. A study was excluded if it was a case report, editorial, letter, or review. If there was disagreement, the full report was retrieved and an independent assessment was repeated until consensus was reached.

### Data abstraction

The design of data abstraction forms and evidence tables were guided by the questions in the analytic framework (Online Resource 2) and approved and finalized by the Skin Bioburden Reduction working group at Canadian Blood Services (through JM). Two reviewers (AG and AD) independently collected data for the following study characteristics: first author, year of publication, country, sample size, donor type, recovery site, tissues collected, pre-recovery skin disinfection method, post-recovery storage parameters and preservation methods. The bioburden testing method was summarized for each study. Data collected relating to the outcomes included: microbes detected immediately following tissue recovery, bioburden immediately following tissue recovery and disinfection, antimicrobial intervention following bioburden assessment, incubation parameters, tissue integrity, and the proportion of allografts discarded due to contamination.

### Quality assessment

There were no clinical studies found among the final pool of included articles. There is no validated quality assessment tool for laboratory-based studies, such as Grading of Recommendations Assessment, Development and Evaluation (GRADE) (Guyatt et al. 2011). Basic science research is inherently considered level IV, or low quality evidence (Balshem et al. 2011).

### Data analysis

Data abstracted from all included studies were organized into tables presenting study characteristics, culture methods, and outcomes. Descriptive statistics included the bioburden outcome, the proportion of discarded allografts, and the logarithmic reduction of bioburden. Proportions, means, ranges, and measures of variance such as standard deviations (SD) are presented when available. In laboratory studies, the tissue discard rate is synonymous with the contamination rate. All analyses of these values were performed as one cohort when appropriate.

## Results

### Study selection

**Fig. 1:**
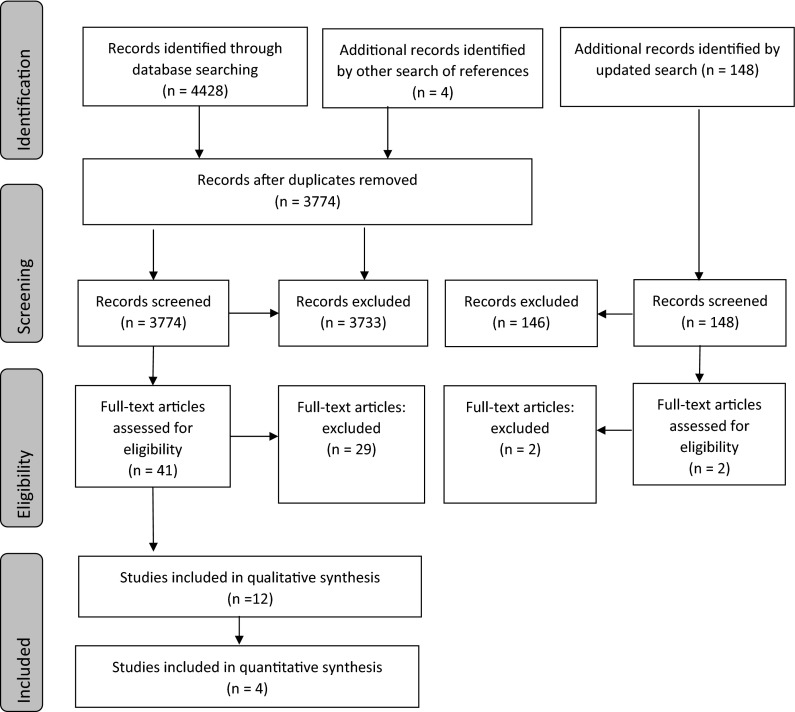
Screening process flow diagram

A total of 3774 citations were reviewed after duplicates were removed (Fig. 1). Of the 3774 records, 3733 were excluded because they did not fulfill the eligibility criteria. The full text-articles of the remaining 41 citations were retrieved for further evaluation. Twenty-nine studies were excluded for varying reasons as listed in Online Resource 3. Four additional citations were added that were identified by searches of the reference lists of the included articles. Following the updated search (2014–2015), an additional 148 articles were reviewed, and 2 were identified for further evaluation. Both articles were excluded due to the lack of meeting the bioburden outcome criteria. Overall, twelve papers met the inclusion criteria.

### Characteristics and culture methods of the studies

All 12 studies were observational and were conducted from 1994 to 2012, with the majority in Europe (58.3 %, 7/12; Table 1). Studies were all single-centered laboratory reports. Six studies were prospective (Baldeschi et al. 1998; Kairiyama et al. 2009; Lomas et al. 2003; Rooney et al. 2008; van Baare et al. 1998; White et al. 1991) and six were retrospective in design (Ireland and Spelman 2005; Lindford et al. 2010; Mathur et al. 2009; Neely et al. 2008; Pianigiani et al. 2010; Pirnay et al. 2012).

**Table 1:** Study characteristics

References	Country	Sample size	Donor^a^	Recovery site	Tissues
Pirnay et al. (2012)	Belgium	148 Donors	Cadaveric	Operating theatre or autopsy room	Skin allografts
Lindford et al. (2010)	Netherlands	115 Donors	Organ	Operating theatre	Skin allografts
Pianigiani et al. (2010)	Italy	723 Donors	Cadaveric organ living	Operating theatre	Skin allografts
Kairiyama et al. (2009)	Argentina	106 Skin batches	Cadaveric	NR	Skin allografts
Mathur et al. (2009)	India	30 Skin grafts	Cadaveric	NR	Skin allografts
Neely et al. (2008)	USA	114 Skin grafts	NR	Operating room	Skin allografts
Rooney et al. (2008)	UK	3 Donors	NR	NR	Skin allografts
Ireland and Spelman (2005)	Australia	534 samples from 24 donors	NR	NR	Musculoskeletal, skin and cardiac allografts
Lomas et al. (2003)	UK	4 Donors	Cadaveric	NR	Skin allografts
Baldeschi et al. (1998)	UK	40 Skin grafts	NR	NR	Skin allograft (eyelid)
van Baare et al. (1998)	Netherlands	1929 Donors	Cadaveric	Autopsy room	Skin allograft
White et al. (1991)	USA	182 Samples from 19 donors	Cadaveric	Autopsy room	Skin allografts

*NR* not reported

^a^Samples were recovered from cadavers (tissue donor), organ (organ and tissue) or from living patients

A total of 2965 donors, and 2575 allografts/homografts and skin samples were reported in twelve studies. In seven studies, skin samples were harvested from cadaveric human donors. In other studies, samples were harvested from a combination of cadaveric, organ, and living donors (Pianigiani et al. 2010), or were harvested solely from organ donors (Lindford et al. 2010). Prior to sample procurement, shaving of the area was performed in three studies before rinsing and cleaning with a variety of agents, including 7.5 % polyvidone-iodine soap, 0.5 % (weight/volume, w/v) chlorhexidine with 70 % isopropanol solution, betadine scrub, or Dodosep (Online Resource 4). The presence of microorganisms was confirmed by culturing of bacterial or fungal species (Online Resource 5). Ten studies reported that 75.0 % (1931/2575) of allograft samples were eligible for release and/or were culture negative for microbial contamination prior to additional disinfection (Baldeschi et al. 1998; Ireland and Spelman 2005; Lindford et al. 2010; Mathur et al. 2009; Neely et al. 2008; Pianigiani et al. 2006; Pirnay et al. 2012; Rooney et al. 2008; van Baare et al. 1998; White et al. 1991).

Following procurement, skin grafts were stored in cold phosphate buffer saline (PBS) at 2–10 °C and pH 7.2–7.3 for 24–72 h (Lomas et al. 2003; Pianigiani et al. 2010; Pirnay et al. 2012; Rooney et al. 2008). Prior to long-term storage, samples were preserved using cryopreservation at −80 to −135 °C, or through glycerol preservation.

An antimicrobial intervention was included in all 12 studies to reduce contamination of the allograft by harmful pathogens. Interventions included incubation with antibiotic/antifungal agents (containing penicillin, streptomycin, amphotericin B, gentamicin, imipenem, polymyxin B, vancomycin, nystatin, amikacin, or ceftazimidine), irradiation, or incubation with peracetic acid (PAA) or glycerol (Online Resource 6).

### Study outcomes

#### Contamination rate and microbe identification

Prior to processing/disinfection of tissues, the contamination rate ranged from 10.1 to 95 % of the allograft samples containing one or more positive result for microorganisms (mean 37.2 %, SD 32.3 %). Ten studies cleaned and disinfected the skin tissue prior to recovery of the allograft. No difference in the contamination rate was observed between disinfection agents.

The most common microorganisms observed following tissue recovery were *Staphylococcus* and *Propionibacterium* species (commensal skin flora). Other organisms cultured from the allografts included *Acinetobacteria, Streptococcus epidermidis, Micrococcus, Candida albicans, Bacillus spp.,* and *Escherichia coli*.

#### Contamination rate reduction

The greatest reduction in contamination and allograft discard rate with regards to antibiotic treatments occurred when the allografts were incubated with three different combinations of antibiotics: (1) penicillin (100 U/ml), streptomycin (100 units (U)/ml), and amphotericin B (1.25 µg/ml) (Pirnay et al. 2012); (2) penicillin (100 U/ml) and streptomycin (100 U/ml) in 85 % glycerol (Pianigiani et al. 2010); and (3) penicillin, streptomycin, kanamycin, gentamicin, and nystatin (Neely et al. 2008). These antibiotic combinations resulted in the greatest reduction of the allograft discard rate (1.4, 1.2 and 0 %, respectively). The tissue discard rate was 0 % in one study despite identifying a positive culture rate of 6.8 % of tissues following disinfection (Neely et al. 2008). The use of streptomycin and penicillin with glycerol or cryopreservation were effective in reducing the initial disinfection rate from an average of 29.6–9.6 % among five studies. In the studies that used antibiotics to disinfect tissues, the allograft discard rate ranged from 2.8 to 35 %. Incubation times ranged from 3 h to 4 weeks at temperatures between 2 and 38 °C. The addition of amphotericin B (a fungicide) to the penicillin and streptomycin antibiotic mixture reduced the contamination rate to only 47.4 % in one study (Pirnay et al. 2012).

In addition to antibiotics, other studies used radiation or chemical agents for allograft disinfection. The use of radiation demonstrated 100 % disinfection of bacterial monoculture after exposure to an average of 25 kGy (Kairiyama et al. 2009). Irradiation of samples at ambient versus frozen temperatures showed no difference in effectiveness, and were able to achieve a > 8 fold log reduction in bioburden with 25 kGy irradiation treatment in 20 and 50 % glycerol solutions (Rooney et al. 2008). In another study, treatment of samples with PAA (0.1 %) demonstrated 100 % disinfection in all samples after a 90 min incubation period (Lomas et al. 2003).

#### Tissue integrity

Two studies performed tissue integrity tests on allografts exposed to different disinfection methods (Lomas et al. 2003; Rooney et al. 2008). Tissue integrity (intact epidermis, papillary and retdicular dermis) was maintained after irradiation when samples were stored in either 20 of 50 % glycerol in PBS (v/v) (Rooney et al. 2008). Processing of tissues in 0.1 % PAA did not affect tissue integrity whereas grafts treated with PAA and preserved in propylene glycol were at risk for collagenase digestion compared to untreated controls (Lomas et al. 2003). Tissue integrity tests following antibiotic treatments were not reported in any of the remaining studies.

#### Confounding factors

The addition of antibiotics and other disinfection methods decrease the contamination and allograft discard rate, but there is little consensus as to the combination, type and concentration of antibiotics used, as well as the optimal solutions to protect tissue undergoing irradiation. For example, in conjunction with other antibiotics, the quantity of penicillin added ranged from 100 to 1000 U/ml and from 1 to 30 µg/ml. The incubation environment during the antibiotic treatment varied greatly. Incubation temperatures ranged from 2 to 38 °C, whereas the incubation period was as short as 3 h, and extended up to 4 weeks.

## Discussion

In this review, contamination of tissues following recovery was found to be quite variable despite attempts to reduce bioburden prior to recovery, such as the method used to disinfect the skin. Processes to disinfect tissues with a combination of antibiotics and antifungals, irradiation or PAA treatment were found to be effective in reducing positive culture results and have minimal, if any effect on tissue integrity. Preservation of samples with 10 or 20 % glycerol compared to other methods of cryopreservation was shown to have an antimicrobial effect.

Following recovery of the skin allografts, it was found that between 10.1 and 95 % of the allografts were culture positive. When reported, different methods of pre-recovery skin preparation did not appear to reduce the contamination rate. All 12 studies disinfected all samples/allografts, regardless of the level of contamination. Among all studies, the average contamination rate was reduced from 37.2 to 15.3 % positive tissue following intervention.

The most common disinfection strategy to reduce the bioburden in studies was a combination of broad spectrum antibiotics with antifungal agents. In three studies, the tissue discard rate was reduced to an average of 19.1 % when penicillin and streptomycin were used in conjunction with the antifungal agent, amphotericin B or nystatin (Pianigiani et al. 2010; Pirnay et al. 2012; White et al. 1991). The presence of the fungus, *Candida,* was observed in the initial bioburden following tissue recovery in five studies, suggesting their relatively consistent prevalence in skin allografts and the potential need to address these contaminating fungi when choosing a disinfection procedure (Baldeschi et al. 1998; Ireland and Spelman 2005; Mathur et al. 2009; Pianigiani et al. 2010; Pirnay et al. 2012). The clinical use of antibiotics and antifungals clinically to treat infections attests to their relative safety when being applied to tissue in relation to cellular integrity. However, tissue integrity was not assessed in any of these studies.

The studies differed in the concentration of antibiotics used (when reported), as well as the incubation duration and temperature at which disinfection occurred. Minimal differences in bioburden reduction were observed with varying concentrations of antibiotics, but it was found that incubation of the tissue with antibiotics at a temperature of 37 °C for 3 h was effective in reducing the number of positive cultures to 13.7 % (ranging from 0 to 27.4 %) in two studies (Ireland and Spelman 2005; Lindford et al. 2010). Generally, antibiotic activities are greatest at 38 °C, and decrease with temperature (Lindford et al. 2010). Most studies chose to use a lower temperature for an extended period of time (up to 4 weeks) to inhibit bacterial growth. Incubation of tissues in the antibiotic-containing solution at 4 °C for 4 weeks reduced the contamination rate from 26.6 to 1.24 % in one study (Pianigiani et al. 2010). Interestingly, incubation at the same temperature for an intermediate period of 1–6 days showed a higher proportion of samples with positive culture, averaging 23.2 % (Baldeschi et al. 1998; Lomas et al. 2003; Mathur et al. 2009; Pirnay et al. 2012; White et al. 1991).

One study utilized PAA to disinfect the allografts. Lomas et al. (2003) treated the tissues with 0.1 % PAA and were able to achieve 100 % reduction in contamination rate following a 90 min incubation, with no observable impact on tissue integrity (Lomas et al. 2003). PAA is advantageous in that it readily kills almost all bacteria, including spores. Its breakdown products (oxygen and acetic acid) are harmless to humans, and it can be effectively used to cold-sterilize temperature sensitive equipment and tissues that are not amenable to heat sterilization or incubation at high temperatures (Lomas et al. 2003).

Irradiation was evaluated in two studies and found that 25 kGy is sufficient for disinfection of skin allografts (Kairiyama et al. 2009; Rooney et al. 2008). An up to 5.2-fold logarithmic reduction (complete disinfection) in bioburden was observed after exposure to up to 33.4 kGy (Kairiyama et al. 2009). The authors note that establishment of a standard irradiation level (such as 25 kGy seen in Rooney et al. 2008), will require a method to reduce the bioburden to acceptable levels (number of microorganisms in the allograft are lower than the logarithmic reduction potential of the disinfection method) prior to irradiation. The sterility assurance level or the probability of a contaminating microorganism following disinfection is extremely dependent on the initial bioburden (Rooney et al. 2008). In other reported studies, irradiation may reduce tissue integrity at higher doses, but irradiation of the tissues while stored in 20 % glycerol solutions provides a protective effect, greatly reducing the incidence of tissue damage (Rooney et al. 2008).

Tissue banks worldwide use cryopreservation (storage at <−150 °C) to maintain tissue integrity over long periods. High concentrations (70–85 %) of glycerol have been reported to be bactericidal (Lindford et al. 2010). When penicillin and streptomycin were solely used to disinfect allografts, the average proportion of positive cultures was 22 % (Ireland and Spelman 2005; Mathur et al. 2009). In other studies, the addition of glycerol preservation to the penicillin and streptomycin treatment reduced the average contamination rate to 0.4 % (Lindford et al. 2010; Pianigiani et al. 2010; van Baare et al. 1998). The addition of glycerol to antibiotic treatment appears to greatly reduce the incidence of contamination. However, the suitability of glycerol preservation for long term storage, or the financial implications of this technique were not reported in these studies (Lindford et al. 2010; Pianigiani et al. 2010; van Baare et al. 1998).

## Limitations

Integrity of the allografts following disinfection was reported in only two articles, but is extremely important for a successful allograft. In addition, transplantation outcomes were not reported in any of these studies.

The contamination rate was determined using standard microbiological culturing techniques on a variety of different culture media and conditions. Although this is the standard practice among labs worldwide, the choice of medium and incubation conditions are extremely important, as many bacteria and fungi can only grow within a very limited set of conditions. A sensitive assay to culture all organisms (or at least pathogenic organisms) is extremely important in reducing the incidence of transplantation-associated infections. The study methods were highly variable, and the optimal method of bacterial identification is not known.

The identification of the bacterial and fungal species is also equally important. Of the identified organisms in these reports, many of these species would not be considered pathogenic. However, patients receiving allografts may be immunocompromised, and the impact of contamination with normal skin flora is unknown. The classification of pathogenicity is relative, and will determine the suitability for the allograft to be released for transplantation. In these laboratory studies, all tissues with contaminating microorganisms (positive culture) would hypothetically be discarded and not used as allografts. Interestingly, Neely et al. (2008) suggested that all of their allografts could be released for transplantation, despite 6.8 % of allografts having a positive culture result. They noted that all contaminating organisms were part of normal skin flora, but quantification of the bioburden was not performed.

Evaluation of the effectiveness of the antimicrobial intervention was performed in this review. The majority of the articles presented the disinfection rate as a proportion of allografts discarded due to contamination or potentially rejected due to positive culture following disinfection. Although this was an effective metric in terms of quality assessments of the antibiotics’ effectiveness in reducing the entire population of microorganisms, a more quantitative method of bioburden would allow for more accurate and optimized recommendations. In this review, the bioburden or bacterial load was only reported in three articles (Lomas et al. 2003; Pirnay et al. 2012; Rooney et al. 2008). The reduction in bioburden provides the greatest wealth of information regarding the effectiveness of the antimicrobial treatment, as the effects of the intervention are easily quantified. Therefore, the majority of the recommendations in this review are based on the reduced rate of contamination (proportion of allograft samples that are culture positive following disinfection).

## Conclusion

The results of this systematic review found that the use of broad spectrum antibiotics in conjunction with antifungal agents, at low temperatures, disinfection with 0.1 % PAA, or with 25 kGy of gamma irradiation results in a reduction of the tissue contamination rate, as opposed to the use of broad spectrum antibiotics alone. Disinfection of the skin prior to recovery is presumed to be important, but the use of common cleansers such a chlorhexidine, povidone-iodine, and Dodesept all appear to have similar efficacies in reducing bioburden following tissue recovery. Given that the studies in this review did not test the efficacies of antimicrobial interventions relative to one another, and are mostly relegated to laboratory studies (where transplantation of the treated tissues is not performed and evaluated for its clinical effectiveness), these outcomes should be interpreted with caution.

## Electronic supplementary material

Below is the link to the electronic supplementary material.

Supplementary material 1 (PDF 25 kb)

Supplementary material 2 (PDF 37 kb)

Supplementary material 3 (PDF 73 kb)

Supplementary material 4 (DOCX 15 kb)

Supplementary material 5 (PDF 80 kb)

Supplementary material 6 (PDF 168 kb)
