# Supplementary material for: Disinfection of human skin allografts in tissue banking: a systematic review report
Source: Cell Tissue Bank. 2016 Aug 13;17(4):585–92. doi: 10.1007/s10561-016-9569-2 (PMC5116035; doi:10.1007/s10561-016-9569-2)
Supplement: Supplementary file 1 — Supplementary material 1 (PDF 25 kb) [file 10561_2016_9569_MOESM1_ESM.pdf]

Online Resource 1: Search Strategies

MEDLINE

1	Skin Transplantation/
2	((skin? or derm*) adj2 transplant*).mp.
3	((skin? or derm*) adj2 (graft* or isograft* or iso-graft* or allograft* or allo-graft* or autograft* or auto-graft* or autogeneicgraft* or autogeneic-graft* or syngraft* or syn-graft* or syngeneicgraft* or syngeneic-graft* or homograft* or homo-graft*)).mp.
4	(dermatoplast* or dermato-plast*).mp.
5	or/1-4
6	Cryopreservation/
7	Tissue Preservation/
8	Organ Preservation Solutions/
9	Tissue Banks/
10	exp Cryoprotective Agents/
11	exp Anti-Bacterial Agents/
12	exp Antifungal Agents/
13	exp Sterilization/
14	exp Pressure/
15	exp Temperature/
16	Time Factors/
17	Transportation/
18	(cryopreserv* or cryo-preserv*).mp.
19	((tissue? or skin) adj2 preserv*).mp.
20	(tissue? adj2 solution?).mp.
21	(tissue? adj2 (bank or banking)).mp.
22	(skin adj2 (bank or banking)).mp.
23	(cryoprotective adj2 agent?).mp.
24	anti-bacterial agent?.mp.
25	antibacterial agent?.mp.
26	bacteriocid*.mp.
27	antifung* agent?.mp.
28	anti-fung* agent?.mp.
29	fungicid*.mp.
30	(steriliz* or sterilis*).mp.

31 pressure*.mp.
32 temperature?.mp.
33 rinsate*.mp.
34 (rinse* or rinsing).mp.
35 time factor?.mp.
36 (incubat* adj2 time).mp.
37 (recover* adj2 time).mp.
38 (ischem* adj2 time).mp.
39 transport*.mp.
40 storage*.mp.
41 (processing adj2 media).mp.
42 (bioburden or bio-burden).mp.
43 or/6-42
44 5 and 43
45 exp animals/ not (exp humans/ and exp animals/)
46 44 not 45
47 limit 46 to (clinical conference or congresses or consensus development conference or consensus development conference, nih)
48 46 not 47
49 limit 48 to (english language and yr="1988 -Current")

EMBASE

1 skin transplantation/
2 exp skin graft/
3 ((skin? or derm*) adj2 transplant*).mp.
4 ((skin? or derm*) adj2 (graft* or isograft* or iso-graft* or allograft* or allo-graft* or autograft* or auto-graft* or autogeneicgraft* or autogeneic-graft* or syngraft* or syn-graft* or syngeneicgraft* or syngeneic-graft* or homograft* or homo-graft*)).mp.
5 (dermatoplast* or dermato-plast*).mp.
6 or/1-5
7 cryopreservation/
8 tissue preservation/
9 skin preservation/
10 exp preservation solution/
11 "preservation and storage"/

12 skin graft storage/
13 cryoprotective agent/
14 exp antifungal agent/
15 exp pressure/
16 temperature/
17 incubation time/
18 cold ischemia/
19 (cryopreserv* or cryo-preserv*).mp.
20 ((tissue? or skin) adj2 preserv*).mp.
21 (tissue? adj2 solution?).mp.
22 (tissue? adj2 (bank or banking)).mp.
23 (skin adj2 (bank or banking)).mp.
24 (cryoprotective adj2 agent?).mp.
25 anti-bacterial agent?.mp.
26 antibacterial agent?.mp.
27 bacteriocid*.mp.
28 antifung* agent?.mp.
29 anti-fung* agent?.mp.
30 fungicid*.mp.
31 (steriliz* or sterilis*).mp.
32 pressure*.mp.
33 temperature?.mp.
34 rinsate*.mp.
35 (rinse* or rinsing).mp.
36 time factor?.mp.
37 (incubat* adj2 time).mp.
38 (ischem* adj2 time).mp.
39 (recover* adj2 time).mp.
40 transport*.mp.
41 storage*.mp.
42 (processing adj2 media).mp.
43 (bioburden or bio-burden).mp.
44 or/7-43
45 6 and 44

46	(exp animals/ or exp animal experimentation/) not ((exp animals/ or exp animal experimentation/) and exp human/)
47	45 not 46
48	limit 47 to (conference abstract or conference paper or conference proceeding or "conference review")
49	47 not 48
50	limit 49 to (english language and yr="1988 -Current")
51	limit 50 to embase
