# Supplementary material for: Disinfection of human skin allografts in tissue banking: a systematic review report
Source: Cell Tissue Bank. 2016 Aug 13;17(4):585–92. doi: 10.1007/s10561-016-9569-2 (PMC5116035; doi:10.1007/s10561-016-9569-2)
Supplement: Supplementary file 2 — Supplementary material 2 (PDF 37 kb) [file 10561_2016_9569_MOESM2_ESM.pdf]

Online Resource 2: Analytical Framework

Cryopreserved Skin

1. What Cryopreserved Skin processing method, or combination of methods, will be the most effective in reducing the bioburden content (quantitative load) while maintaining tissue quality (the ability of the tissue to function as intended)?
    a. What are the advantages and disadvantages of each method?
        i. Antibiotic selection?
        ii. Antifungal selection?
        iii. Antibiotic and or antifungal incubation?
        iv. Cleaning and rinsing?
        v. Storage and transportation?
        vi. Irradiation?
        vii. Non antibiotic based chemical disinfection or sterilization?
        viii. Super critical CO_2?

    b. What antibiotic combination is optimal for reducing the bioburden content (load) Optimal is defined as the highest log reduction in bioburden. A six log reduction is considered sterile.

    c. What incubation parameters maximize the reduction of bioburden content (load)?
        i. Temperature?
        ii. Timing of initiation (i.e. immediately post recovery)?
        iii. Duration?
        iv. Physical conditions such as pressure, agitation, vacuum or sonication?
        v. PH?

    d. What cleaning and rinsing processes are most effective in reducing bioburden content (load)?
        i. Rinsing solution?
        ii. Rinsing parameters / reagent removal?
        iii. Assessing residual cytotoxicity?
        iv. Testing for pyrogens?

    e. What preservation is most effective in reducing bioburden content (load)?
        i. Cryopreservation – controlled rate freeze
        ii. Cryopreservation - heat Sink
        iii. Glycerol
        iv. Freeze Drying
        v. Ethanol Storage

f. What storage and transportation parameters are most effective in reducing bioburden content/load?
    i. Storage method?
    ii. Storage temperature?
    iii. Transportation process?

2. In wound care patients for skin graft transplantation, does skin graft processed on a specific method or combination result in increased graft survival and/or reduction in mortality/infection/co-morbidities?

3. What is the prevalence of wound care patients in need of a skin graft?
    a. What proportion of patients receives a skin graft to increase survival and decrease morbidities?
    b. Are there patients at risk of failure or success of transplant? Risks refer to relevant patient data.

Fresh Skin Grafts

1. What Fresh Skin processing method, or combination of methods, will be the most effective in reducing the bioburden content (load) while maintaining tissue quality (the ability of the tissue to function as intended)?
    a. What are the advantages and disadvantages of each method?
        i. Antibiotic selection?
        ii. Antifungal selection?
        iii. Cleaning and rinsing?
        iv. Storage and transportation?
        v. Non-antibiotic based disinfection?

    b. What antibiotic combination is optimal for reducing the bioburden content (load). Optimal is defined as the highest log reduction in bioburden. A six log reduction is considered sterile.

    c. What incubation parameters maximize the reduction of bioburden content (load)?
        i. Temperature?
        ii. Timing of initiation (i.e. immediately post recovery)
        iii. Duration?
        iv. Physical conditions such as pressure, agitation, vacuum or sonication?
        v. PH?

    d. What cleaning and rinsing processes are most effective in reducing bioburden content (load)?
        i. Rinsing solution?
        ii. Rinsing parameters – reagent removal?

iii. Assessing residual cytotoxicity
iv. Testing for pyogenes?

e. What storage and transportation parameters are most effective in reducing bioburden content (load)?
   i. Storage method?
   ii. Storage solution (glycerol)
   iii. Storage temperature?
   iv. Transportation process?
   v. Duration – what is the maximum length of storage?
