# Supplementary material for: Disinfection of human skin allografts in tissue banking: a systematic review report
Source: Cell Tissue Bank. 2016 Aug 13;17(4):585–92. doi: 10.1007/s10561-016-9569-2 (PMC5116035; doi:10.1007/s10561-016-9569-2)
Supplement: Supplementary file 3 — Supplementary material 3 (PDF 73 kb) [file 10561_2016_9569_MOESM3_ESM.pdf]

Online Resource 3: Excluded Studies

First Author, Year	Reason for exclusion
Bianco, 2014	No analysis of bioburden
Bourroul 2002	No analysis of bioburden
Britton-Byrd 2008	No decontamination method
Caemeron 2000	HIV kinetics in an experimental setup
deBackere 1994	Review of European practice: no bioburden outcome
Eastland 2006	Case reports
Fielding 1988	Review article
Gala, 1997	Did not analyze bacterial bioburden
Ghosh 1994	Letter to editor
Hermans 2011	Review article
Herson 2009	Impact of IAEA program Brazil
Holder 1999	Assay method; no tissue
Huang 2004	No bioburden outcome
Kearney 2005	Guideline
Lomas 2004	No bioburden outcome
Mackie 2002	Survey of skin preservation: no bioburden outcome
Marshall 1995	Experimental model in cultured human fibroblast
Mericka 2006	Review of standards: no bioburden outcome
Morales Pedraza 2009	No bioburden outcome
Peralam 2011	Letter
Pianigiani 2006	No decontamination method
Pirnay 1997	Literature review HIV transmission in skin. No bioburden outcome
Richters 1996	Morphology following preservation: no bioburden outcome
Saegeman 2007	Bacterial integrity analyzed in an experimental set-up
Titley 1994	No decontamination method or bioburden outcome
Turhan-Haktanir 2011	No bioburden outcome
Van Baare 1994	Did not address bioburden and contamination
Wang 2011	Technical note: no bioburden outcome
Zidan 2014	No bioburden outcome

References of Excluded Studies

Bianco, C., Adami, G., Crosera, M., Larese, F., Casarin, S., Castagnoli, C., ... & Maina, G. (2014). Silver percutaneous absorption after exposure to silver nanoparticles: A comparison study of three human skin graft samples used for clinical applications. *Burns*, *40*(7), 1390-1396.

Bourroul, S. C., Herson, M. R., Pino, E., & Matho, M. B. (2002). Sterilization of skin allografts by ionizing radiation. *Cellular and molecular biology (Noisy-le-Grand, France), 48*(7), 803-807.

Britton-Byrd, B. W., Lynch, J. P., Williamson, S., & McCauley, R. L. (2008). Early use of allograft skin: are 3-day microbiologic cultures safe?. *Journal of Trauma and Acute Care Surgery, 64*(3), 816-818.

Cameron, P. U., Pagnon, J. C., van Baare, J., Reece, J. C., Vardaxis, N. J., & Crowe, S. M. (2000). Efficacy and kinetics of glycerol inactivation of HIV-1 in split skin grafts. *Journal of medical virology*, *60*(2), 182-188.

de Backere, A. C. (1994). Euro Skin Bank: large scale skin-banking in Europe based on glycerol-preservation of donor skin. *Burns, 20 Suppl 1,* S4-9.

Eastlund, T. (2006). Bacterial infection transmitted by human tissue allograft transplantation. *Cell and Tissue Banking, 7*(3), 147-166.

Fielding, G. A., & Pegg, S. P. (1988). Homograft skin banking--current practices and future trends. *Australian & New Zealand Journal of Surgery, 58*(2), 153-156.

Gala JL, Vandenbroucke AT, Vandercam B, Pirnay JP, Delferrière N, Burtonboy G. (1997) HIV-1 detection by nested PCR and viral culture in fresh or cryopreserved postmortem skin: potential implications for skin handling and allografting. *J Clin Pathol.* 50(6):481-4.

Ghosh, M.M., Freelander E. (1994) Inactivation of viruses by glycerol as used in allograft skin preservation. *Burns*,20(5):476-7.

Hermans, M. H. (2011). Preservation methods of allografts and their (lack of) influence on clinical results in partial thickness burns. *Burns*, *37*(5), 873-881.

Herson, M. R., Mathor, M. B., & Pedraza, J. M. (2009). The impact of the International Atomic Energy Agency (IAEA) program on radiation and tissue banking in Brazil. *Cell and tissue banking*, *10*(2), 143-147.

Holder, I. A., Robb, E., & Kagan, R. (1998). Antimicrobial mixtures used by tissue banks for harvested skin: comparative in vitro activity. *Burns, 24*(7), 604-608.

Huang, Q., Pegg, D. E., & Kearney, J. N. (2004). Banking of non-viable skin allografts using high concentrations of glycerol or propylene glycol. *Cell and tissue banking*, *5*(1), 3-21.

Kearney, J. N. (2005). Guidelines on processing and clinical use of skin allografts. *Clinics in dermatology*, *23*(4), 357-364.

Lomas, R. J., Huang, Q., Pegg, D. E., & Kearney, J. N. (2004). Application of a high-level peracetic acid disinfection protocol to re-process antibiotic disinfected skin allografts. *Cell and tissue banking*, *5*(1), 23-36.

Mackie, D. (2002). Postal survey on the use of glycerol-preserved allografts in clinical practice. *Burns, 28 Suppl 1*, S40-44.

Marshall, L., Ghosh, M. M., Boyce, S. G., MacNeil, S., Freedlander, E., & Kudesia, G. (1995). Effect of glycerol on intracellular virus survival: implications for the clinical use of glycerol-preserved cadaver skin. *Burns,21*(5), 356-361.

Mericka, P. (2006). Current trends in safety assurance for tissue grafts used in burn treatment. *Acta Chirurgiae Plasticae, 48*(2), 51-58.

Pedraza, J. M., & Phillips, G. O. (2009). The impact of the International Atomic Energy Agency (IAEA) program on radiation and tissue banking in Asia and the Pacific and the Latin American regions. *Cell and tissue banking, 10*(2), 79-86.

Peralam, P. Y. (2011). Letter: Some Caveats in Skin Bank Practices from the Perspectives of Fungal Skin Flora. *International wound journal, 8*(2), 211-211.

Pianigiani, E., Risulo, M., Ierardi, F., Sbano, P., Andreassi, L., Fimiani, M., ... & Zazzi, M. (2006). Prevalence of skin allograft discards as a result of serological and molecular microbiological screening in a regional skin bank in Italy. *Burns, 32*(3), 348-351.

Pirnay JP , CVandenvelde C HIV transmission by transplantation of allograft skin: a review of the literature Burns 1997:23:1-5

Richters, C. D., Hoekstra, M. J., van Baare, J., du Pont, J. S., & Kamperdijk, E. W. (1996). Morphology of glycerol-preserved human cadaver skin. *Burns, 22*(2), 113-116.

Saegeman, V., Lismont, D., Verduyckt, B., Ectors, N., Stuyck, J., & Verhaegen, J. (2007). Antimicrobial susceptibility of coagulase-negative staphylococci on tissue allografts and isolates from orthopedic patients.*Journal of orthopaedic research, 25*(4), 501-507.

Titley, O. G., Cooper, M., Thomas, A., & Hancock, K. (1994). Stored skin--stored trouble? *British Journal of Plastic Surgery, 47*(1), 24-29.

Turhan-Haktanir, N., Dilek, F. H., Koken, G., Demir, Y., & Yilmaz, G. (2011). Evaluation of amniotic fluid as a skin graft storage media compared with RPMI and saline. *Burns, 37*(4), 652-655.

Van Baare, J., Buitenwerf, J., Hoekstra, M. J., & Du Pont, J. S. (1994). Virucidal effect of glycerol as used in donor skin preservation. *Burns, 20*, S77-S80.

Wang, Y. Y., He, J. Y., Liu, X. P., & Li, X. F. (2011). A reliable stowage by means of syringe for preserving split-thickness-skin grafts. *Journal of Tissue Viability, 20*(1), 35-36.

Zidan, S. M., & Eleowa, S. A. (2014). Banking and use of glycerol preserved full-thickness skin allograft harvested from body contouring procedures.*Burns*, *40*(4), 641-647.
