# Supplementary material for: Disinfection of human skin allografts in tissue banking: a systematic review report
Source: Cell Tissue Bank. 2016 Aug 13;17(4):585–92. doi: 10.1007/s10561-016-9569-2 (PMC5116035; doi:10.1007/s10561-016-9569-2)
Supplement: Supplementary file 4 — Supplementary material 4 (DOCX 15 kb) [file 10561_2016_9569_MOESM4_ESM.docx]

**Online Resource 4: Recovery and Storage Parameters**

| **First Author,**  **Year** | **Pre-Recovery Skin Preparation** | **Post-Recovery Storage Parameters** | **Preservation Method** |
| --- | --- | --- | --- |
| Pirnay 2012 | Shave and thoroughly scrub with 7.5% povidone–iodine soap, rinse with tap water and 0.5% (w/v) chlorhexidine with 70% (v/v) isopropanol solution | Placed in transport media, transported on crushed ice and stored at 2-8 °C | Cryopreservation |
| Lindford 2010 | Following organ donation the donor is re-scrubbed and draped | NR | Glycerol Preservation |
| Pianigiani 2010 | Clean with betadine scrub, rinse with sterile saline and tincture of chlorhexidine | Storage at +2/+10°C | Glycerol Preservation  Cryopreservation |
| Kairiyama 2009 | NR | NR | Cryopreservation  Storage in 10% Glycerol at  -80˚C |
| Mathur 2009 | 1% povidone–iodine and 70% alcohol | NR | NR |
| Neely 2008 | 4% chlorhexidine and isopropanol | NR | Refrigeration  (+1 – +10˚C)  Cryopreservation |
| Rooney 2008 | NR | Stored in 20% (v/v) glycerol in Hanks Balanced Salt Solution (HBSS, Sigma, Poole, UK) | Cryopreservation  -80°C |
| Ireland 2005 | Skin prep by shaving then two 5 min surgical scrubs using chlorhexidine solution | Medium199 (JRH Biosciences) with 50 µg/ ml streptomycin, 30 µg/ml penicillin | Cryopreservation (Vapour phase nitrogen,  -135˚C to -190˚C) |
| Lomas 2003 | Sequential applications of a surgical scrub (Hydrex, Adams), 10% (w/v) povidine iodine solution and chlorhexidine–alcohol spray | Storage in PBS at (4–10°C),  pH 7.2–7.3 | NR |
| Baldeschi 1998 | Skin was decontaminated with povidone-iodine  solution (100 mg/ml) | 4°C for 6 days in sterile saline | NR |
| van Baare 1998 | Cleaned with betadine scrub, and 0.5% chlorhexidine digluconate in 70% ethanol | 4°C | Glycerol preservation |
| White 1991 | Skin is shaved and scrubbed with povidone-iodone solution for 5-10 minutes, then rinsed with water. Surface is painted with povidone-iodine solution again, allowed to dry, then rinsed with 70% isopropyl alcohol | Stored in 10% fetal bovine serum in Dulbecco’s modified Eagle medium (DME) with 5% antibiotic/anti-mycotic solution (penicillin, streptomycin, and amphotericin B) | Cryopreservation  Preserved in DME with 10% fetal serum and 10% glycerol and step-wise cooled to -90°C, and stored in vapour phase of liquid nitrogen. |

NR = not reported; PBS= phosphate buffer solution; w/v = weight/volume
