# Supplementary material for: Disinfection of human skin allografts in tissue banking: a systematic review report
Source: Cell Tissue Bank. 2016 Aug 13;17(4):585–92. doi: 10.1007/s10561-016-9569-2 (PMC5116035; doi:10.1007/s10561-016-9569-2)
Supplement: Supplementary file 5 — Supplementary material 5 (PDF 80 kb) [file 10561_2016_9569_MOESM5_ESM.pdf]

Online Resource 5: Identification Methods

First Author, Year	Identified Organism	Bioburden Testing Method
Pirnay 2012	Bacteria	20 randomly selected skin pieces of approximately 2 cm^2 (1–2% of the skin donation) were obtained aseptically and transferred to a receptacle with 25 ml of thioglycollate broth with resazurin and to a recipient with 25 ml of Sabouraud broth.
Lindford 2010	Bacteria	The first bacterial culture of the allografts is taken three weeks after procurement. If after three weeks of refrigeration a positive culture is obtained the skin is placed under quarantine and re-cultured immediately for secondary testing.
Pianigiani 2010	Bacteria Fungi	A 50 ml aliquot of homogenized skin cells is inoculated in 1) generic medium for aerobes (Columbia blood and chocolate agar Plates—BioMerieux) at 37˚C for 48 h, 5%CO_2 2) generic medium for anaerobes (Schaedler agar plates—BioMerieux) at 37˚C for 72 h in anaerobic conditions and 3) medium for slow-growing bacteria (7H11 BecktonDickinson) at 37˚C for 30 days, 5% CO_2. Inoculation of samples into Sabouraud agar with chloramphenicol, incubated at 28°C in air for 21 days allowed for identification of fungi.
Kairiyama 2009	Bacteria	Microorganisms were liberated using Stomacher® machine and suspended in peptone water (0.1% w/v) and filtered through nitro cellulose membranes of 0.45 mm pore size, and cultured on soy bean casein digest agar medium (Difcos), pH7.2, incubated at 32 ± 1°C for five to fourteen days.
Mathur 2009	Bacteria Fungi	To test for bacteria, 1 cm^2s skin fragment was inoculated in glucose broth (incubated at 37˚C for 24 h) for aerobic bacteria, in Sabouraud's dextrose agar (incubated at 37˚C for 7 days) for fungi, and thioglycollate broth (at 37˚C for 48 h) for anaerobic bacteria.
Neely 2008	Bacteria	Tissue samples were cultured after recovery, then after disinfection. Recipient grafts underwent surveillance cultures including throat, nares, stool, all burn sites, and the unburned area next to the burn site. Following this initial set of surveillance cultures, cultures of the burn wounds (allograft sites) are taken twice a week, generally during dressing changes, until the wounds closed
Rooney 2008	Bacteria	Pre-prepared suspension of *Bacillus pumillis* spores (ATCC #27142) was serially diluted into 0%, 20%, 50% 85% glycerol solutions (v/v) Following treatment, 1 ml of the samples were transferred to 20 ml irradiated tryptone soya broth bottles (Cherwell Laboratories) and incubated at 35°C and checked at intervals up to fourteen days.
Ireland 2005	Bacteria	Swabs of bone and tissue samples, as well as cardiac and allograft skin tissues were placed into thioglycollate medium, incubated for 48 h at 35°C.The medium was sub-cultured onto Columbia blood agar plates, and incubated aerobically and anaerobically at 35°C for up to seven days.
Lomas 2003	Bacteria	Serial dilutions of samples were inoculated onto tryptone soya agar (Oxoid) and the number of colonies following incubation were counted.
Baldeschi 1998	Bacteria Fungi	Samples were washed in sterile saline on days 2-6. Serial dilutions of each wash were plated onto blood-agar plates and incubated at 37°C for 24 h.
van Baare 1998	Bacteria	Skin samples were incubated in broth-based glucose medium for 5 days at 37°C and monitored for contamination. If positive, samples were subcultured onto blood agar, cysteine-lactose-electrolyte deficient agar, and chocolate agar medium and grown for 5 days. If no growth, samples were subcultured onto blood agar and chocolate agar under anaerobic conditions to isolate anaerobes.
White 1991	Bacteria	Dilutions of samples were cultured on sheep blood agar, eosin methylene blue and chocolate agar plates and incubated at 37°C for 24-48 hours.

NR = not reported;

Bioburden analysis was performed for the identification of microorganisms following recovery of the tissue. Following antimicrobial intervention (if applicable) of the sample below, the same identification method was used to quantify the remaining number of microorganisms.
