# Supplementary material for: Disinfection of human skin allografts in tissue banking: a systematic review report
Source: Cell Tissue Bank. 2016 Aug 13;17(4):585–92. doi: 10.1007/s10561-016-9569-2 (PMC5116035; doi:10.1007/s10561-016-9569-2)
Supplement: Supplementary file 6 — Supplementary material 6 (PDF 168 kb) [file 10561_2016_9569_MOESM6_ESM.pdf]

Online Resource 6: Study Outcomes

First Author, Year	Microbes Detected Immediately Following Recovery	Bioburden Immediately Following Recovery	Antimicrobial intervention Following Bioburden Assessment	Incubation Parameters	Tissue integrity	Proportion of allografts discarded due to contamination	Logarithmic bioburden reduction
Pirnay 2012	Pathogens (20)<br>*Candida albicans* (5)<br>*Escherichia coli* (3)<br>*Klebsiella pneumonia* (3)<br>*Staphylococcus aureus* (2)<br>*Pseudomona aeruginosa* (2)<br>*Enterococcos faecalis* (2)<br>*Clostridium perfringens* (1)<br>*Enterobacter cloacae* (1)<br>*Enterococcus faecium* (1)<br><br>Non Pathogens (28)	NR	Penicillin (100 U/ml)<br>Streptomycin (100 U/ml)<br>Amphotericin B (1.25 µg/ml)	24-72 h at 2-8 °C	NR	47.4% (27/57) positive cultures<br><br>18 positive <7 day culture incubation<br>9 positive 7-14 day culture incubation	NR
		NR	Gentamicin sulphate (4 mg/ml)<br>Imipenem/cilastin Na (0.2 mg/ml)<br>Polymyxin B (0.2 mg/ml),<br>Vancomycin HCl (0.05 mg/ml)<br>Nystatin (2,500 U/ml)	24-72 h at 2-8 °C	NR	18.7% (17/91) positive cultures<br><br>10 positive > 7day incubation<br>7 positive 7-14 day incubation	NR
		NR	Glycerol Decontamination for samples positive after antibiotic treatment (above)	NR	NR	34.1% (15/44) positive culture	NR
Lindford 2010	Pathogenic (1)<br>*Enterobacter cloacae* (1)<br>Non Pathogens (12)	25% (29/115) positive cultures at 3 weeks storage<br><br>Secondary culture of these 29 were all negative.	85% Glycerol with G-Penicillin and Streptomycin	3 h at 38˚C<br><br>Followed by 85% glycerol, 3h at 38˚C	NR	0% (0/115) of allografts were discarded	NR

First Author, Year	Microbes Detected Immediately Following Recovery	Bioburden Immediately Following Recovery	Antimicrobial intervention Following Bioburden Assessment	Incubation Parameters	Tissue integrity	Proportion of allografts discarded due to contamination	Logarithmic bioburden reduction
Pianigiani 2010	Non-Pathogens<br>*Staphylococcus spp*. (69.0%)<br>*Streptococcus spp*. (1.9%),<br>*Corynebacterium spp*. (4.2%),<br>*Escherichia coli* (1.9%),<br>*Propionibac-terium spp.* (10%),<br>*Micrococcus spp*. (1.9%)<br>*Propionibacterium acnes* (7.1%)<br>Pathogens<br>*Pseudomonas spp*. (1.4%)<br>*Proteus spp.* (0.4%)<br>Mycetes 5.3%<br>Yeasts (73%)<br>*Malassezia spp.*<br>*Candida spp*.<br>*Pseudalescheria boydii* (2/39)<br>*Aspergillus spp.*<br>*Verticillum spp.*<br>*Pennicillium*	26.55% positive cultures (192/723)<br>4.5% (12/265) mycological positivity<br><br>82/192 (42.70%) required corrective actions and 9/82 (11%) remained positive after correction/ disinfection	Glycerol 70%, then add 87% plus (P/S) Penicillin 100 U/ml Streptomycin 100 µg/ml	Incubated at +2/+10°C for 4 wks<br><br>If microbes found after 4 wks antibiogram may be done and antibiotic modified	NR	1.24% (rejected due to gram positive contamination<br><br>Discarded tissues (9/723)<br>*Staphylococcus sp.* (6/9)<br>*Bacillus cereus* (1/9)<br>*Enterococcus faecium* (1/9)<br>*Group G Streptococcus* (1/9)	NR
		26.2% positive culture (120/458)<br>5.8% (27/458) mycological positive culture	Cryopreservative Saline, DMEM, 15% glycerol, Penicillin 100 UI/ml Streptomycin 100 ug.ml, gentamycin 10ug/ml, amphotericin B 5ug/ml	Incubated +in for 4 wks +2/+10°C<br><br>If microbes found after 4 wks antibiogram may be done and antibiotic modified	NR		NR
Kairiyama 2009	NR	<10 to 162700 CFU/100cm^2 (Wide bioburden distribution observed between batches)	17.6 to 33.4 kGy. (Dosage depended on bioburden)	NR	NR	0% positive culture following irradiation.	Logarithmic bioburden reduction ranges from 0 to 5.2 (Starting from <10 to 162700 CFU/ml

<table>
<tr><th>First Author, Year</th><th>Microbes Detected Immediately Following Recovery</th><th>Bioburden Immediately Following Recovery</th><th>Antimicrobial intervention Following Bioburden Assessment</th><th>Incubation Parameters</th><th>Tissue integrity</th><th>Proportion of allografts discarded due to contamination</th><th>Logarithmic bioburden reduction</th></tr>
<tr><td></td><td></td><td></td><td></td><td></td><td></td><td></td><td>and reduced to 0 CFU/ml)</td></tr>
<tr><td rowspan="3">Mathur 2009</td><td>Mix of bacteria and fungi</td><td><u>70% Positive cultures (21/30)</u>

First antibiotic treatment
Bacterial positive cultures 3/21 (14.3%)
Positive fungal culture 7/21 (33.3%)</td><td><u>Grafts with bacteria</u>
First antibiotic treatment
Penicillin 1000U/ml
Gentamicin 800 µg/ml</td><td>4°C for 24 h</td><td>NR</td><td><u>After all 3 treatments, 16.7% (5/30) had positive culture</u>

47.6% positive culture (10/21)</td><td>NR</td></tr>
<tr><td>Mix of bacteria and fungi</td><td>Second antibiotic treatment
Bacterial positive culture 2/10 (20%)</td><td>Secondary Antibiotic treatment
Amikacin 5mg/ml
Ceftazidime
5 mg/ml</td><td>4°C for 24 h</td><td>NR</td><td>90% positive culture (9/10)
Klebsiella and fungal species</td><td>NR</td></tr>
<tr><td>Fungi</td><td>42.8% Positive fungal cultures (3/7)</td><td><u>Grafts with Fungi</u>
Amphotericin B 8mg/100ml</td><td>4°C for 24 h, 48 h and 7 days</td><td>NR</td><td>42.8% (3/7) positive fungal cultures</td><td>NR</td></tr>
<tr><td rowspan="2">Neely 2008</td><td rowspan="2"><u>Commensal non-pathogens</u>
Staphylococcus spp, Proprionibacterium spp, Corynebacterium spp
Bacillus spp.</td><td rowspan="2"><u>19.5% Positive culture prior to treatment (22/114)</u></td><td>Penicillin
Streptomycin
Kanamycin
Gentamicin
Nystatin</td><td>Fresh allografts
1˚C to 10°C for up to 10 days</td><td>NR</td><td rowspan="2">No grafts used clinically.

0% (0/114) allografts were discarded.

6.8% (8/118) positive cultures following treatment</td><td>NR</td></tr>
<tr><td>Penicillin
Streptomycin
Kanamycin
Gentamicin
Nystatin</td><td>Frozen allografts cryopreserved by temperature reduction of -1°C/min to -40°C, then temperature reduction rate of -4°C/min to -100°C</td><td>NR</td><td>NR</td></tr>
</table>

First Author, Year	Microbes Detected Immediately Following Recovery	Bioburden Immediately Following Recovery	Antimicrobial intervention Following Bioburden Assessment	Incubation Parameters	Tissue integrity	Proportion of allografts discarded due to contamination	Logarithmic bioburden reduction
Rooney 2008	*Bacillus pumillis* spores	22.4% Positive culture prior to treatment (20/89)	Serial dilutions of samples were exposed to a minimum 25 kGy while in 0%, 20%, 50% or 85% glycerol.	NR	Samples that were non-irradiated, or irradiated in 20% or 50% glycerol showed epidermis, papillary and reticular dermis) was intact, and stratum corneum attached<br><br>Irradiated samples in 85% glycerol showed abnormal with stratum corneum almost entirely detached, keratinocytes dark and heavily stained.	No samples used as allografts.<br><br>Combined data (not tested) suggest that only skin in 20% glycerol, irradiated frozen, allowed decontamination without any other adverse effects.<br><br>However, there was some indication of cytotoxicity in 50% glycerol.	Treatment with 25 kGy in 20% and 50% glycerol solutions showed >8 fold logarithmic bioburden reduction

First Author, Year	Microbes Detected Immediately Following Recovery	Bioburden Immediately Following Recovery	Antimicrobial intervention Following Bioburden Assessment	Incubation Parameters	Tissue integrity	Proportion of allografts discarded due to contamination	Logarithmic bioburden reduction
		12% Positive cultures prior to treatment (3/25)	Serial dilutions of samples were exposed to a minimum 25 kGy while in 0%, 20%, 50% or 85% glycerol.	Frozen allografts cryopreserved by temperature reduction of -1°C/min to -40°C, then temperature reduction rate of -4°C/min to -100°C	Samples that were irradiated while frozen in 20% or 50% glycerol showed epidermis, papillary and reticular dermis) was intact, and stratum corneum attached<br><br>Irradiated in PBS (0% glycerol) showed clear disruption of dermis with vacuolization of collagen.<br><br>Irradiated samples in 85% glycerol showed abnormal with stratum corneum almost entirely detached, keratinocytes dark and heavily stained		Treatment with 25 kGy in 20% glycerol solutions showed >8 fold logarithmic bioburden reduction
Ireland 2005	Pathogen<br>*Clostridium perfringens*<br><br>Commensal (Non pathogenic)<br>*Staphylococci*<br>*Diphtheroids*<br>*S. aureus*<br>*Bacillus spp*<br>*Propionibacterium acnes*<br>Yeast	15.7 % (84/534) Positive cultures<br><br>85% (72/84) positive cultures (normal skin bacteria)<br><br>10.7% (9/84) Positive samples *Clostridium perfringens* (8/9 isolated from one donor)	Streptomycin (50 µg/ml)<br>Penicillin (30 µg/ml)	35°C for 6–8 h	NR	27.4% (727/2658) discarded<br><br>For skin, musculoskeletal, and cardiac tissue (overall), positive cultures make up 44.2% (898/2031) of rejections	NR

First Author, Year	Microbes Detected Immediately Following Recovery	Bioburden Immediately Following Recovery	Antimicrobial intervention Following Bioburden Assessment	Incubation Parameters	Tissue integrity	Proportion of allografts discarded due to contamination	Logarithmic bioburden reduction
Lomas 2003	*Bacillus subtilis* var. *Niger* (suplemented)	100 %	0.1% PAA	10 minutes<br>20 minutes<br>40 minutes<br>60 minutes<br>90 minutes	Histology intact with 0.35% or 0.1% PAA<br><br>Collagenase susceptibility is increased when using propylene glycol to preserve compared to glycerol)	0% Survival after 90 minute incubation<br><br>No growth seen after 60 minute incubation in reinforced clostridial medium (RCM, Oxoid) medium	*In vitro* studies without tissue show 0.9 ± 0.2 min for 1 log reduction
			0.35% PAA	NR	Not tested on tissue, as previous studies have shown 0.35% PAA reduces tissue integrity	NR	*In vitro* studies without tissue show 2.6 ± 0.1 min for 1 log reduction
Baldeschi 1998	*Staphylococcus epidermidis*<br>*Staphylococcus aureus*<br>*Staphylococcus saprophyticus*<br>*Pseudomonas aeruginosa*<br>*Serratia liquefaciens*<br>*Klebsiella oxytoca*<br>*Candida albicans*	95% positive cultures (38/40)	Gentamicin (2 mg/ml	Up to 6 days at 4°C	NR	35% positive culture after antibiotic treatment (14/40)	NR
van Baare 1998	*Staphylococcus epidermis*<br>*Enterococcus faecalis*<br>*Staphylococcus aureus*<br>*Escehrichia coli*	10.1 ± 4.1% positive culture (n = 1929) from 1987 to 1995	Streptomycin (1 mg/ml)<br>Penicillin G (1000 IU/ml)	At least 2 h at room temperature	NR	0% discarded after 90 days incubation in glycerol with antibiotics (0/1929) (2.8% positive culture after 5 weeks)	NR

First Author, Year	Microbes Detected Immediately Following Recovery	Bioburden Immediately Following Recovery	Antimicrobial intervention Following Bioburden Assessment	Incubation Parameters	Tissue integrity	Proportion of allografts discarded due to contamination	Logarithmic bioburden reduction
	Enterobacter spp.<br>*Bacillus spp.*						
White 1991	*Staphylococcus spp.*<br>*Diptheroids*<br>*Pseudomonas spp*<br>*Enterococcus*	56.6% positive culture (103/182)	Penicillin<br>Streptomycin<br>Amphotericin B<br><br>Dose NR	4°C for 48-192 h	NR	14.8% positive culture after antibiotic treatment. (27/182)	NR

NR = not reported; PAA= peracetic acid

Immediately following recovery of the tissue, microbial detection was performed on the samples using the methods described in Table 2 for bioburden assessment. Using fresh, or previously stored samples, the allografts were treated with the antimicrobial intervention, and tested for integrity/contamination to determine suitability for transplantation. This was reported as the allograft rejection rate.
